# A framework for challenges and solutions in biodesign research^☆^

**DOI:** 10.1016/j.bidere.2025.100029

**Published:** 2025-06-04

**Authors:** Xiaohan Yang, Zhihua Jiang, Shihui Yang, Zong-Ming Cheng

**Affiliations:** Biosciences Division, Oak Ridge National Laboratory, Oak Ridge, TN, 37831, USA; The Center for Bioenergy Innovation, Oak Ridge National Laboratory, Oak Ridge, TN, 37831, USA; Department of Animal Sciences and The Center for Reproductive Biology, Washington State University, Pullman, WA, 99164, USA; State Key Laboratory of Biocatalysis and Enzyme Engineering, and School of Life Sciences, Hubei University, Wuhan, 430062, China; Nanjing Agricultural University, Nanjing, Jiangsu Province, 210095, China

## Main text

The bioeconomy represents an advanced economic paradigm that builds upon previous agricultural, industrial, and digital economic models. It seeks to tackle critical global challenges such as resource scarcity, escalating healthcare demands, and environmental degradation. At the heart of the bioeconomy is biomanufacturing, which uses natural or engineered enzymes or cell factories built from ​biological components like promoters, terminators, regulatory sequences, reporters, and functional genes into various chassis hosts (including animal, microbial, plant, and *de novo* systems) to create products such as food, energy, medicine, materials, chemicals, and engineered tissue/organs. An enabler of biomanufacturing is biodesign – also known as biosystems design and closely related to synthetic biology or engineering biology. This interdisciplinary field aims to understand and predictably modify existing life forms or create entirely new biological entities/systems using rational engineering strategies and automated design tools [1]. Through these capabilities, biodesign supports the discovery, optimization, and creation of efficient platforms for biomanufacturing [2].

**Fig. 1:**
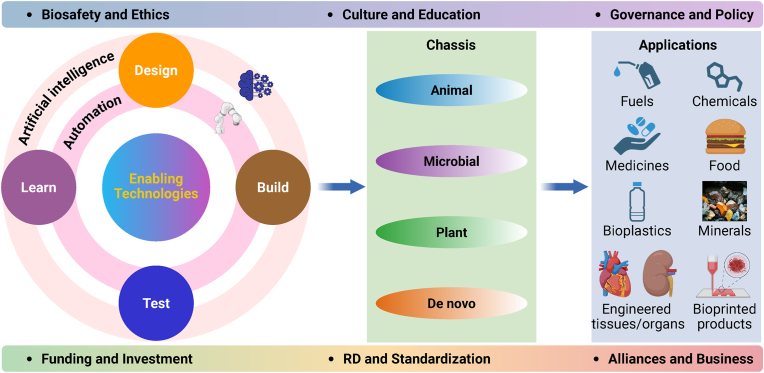
Technological, social, and economic challenges of biodesign on enabling technologies, chassis, and applications. Created with BioRender.com.

As an emerging field, biodesign faces substantial technological, social, and economic challenges (Fig. 1). Key enabling technologies, such as universal biological computer-aided design (bioCAD) software, efficient genome editing and synthesis tools, and high-throughput screening and omics equipment, remain sufficiently immature for reliably designing, building, testing, and optimizing biomanufacturing platforms under real-world industrial conditions [2]. Additionally, the range of available chassis hosts is still largely restricted to a few model species, although interest in non-model species is growing. Despite its vast potential across sectors like energy, environmental sustainability, industry, agriculture, and healthcare, biodesign has achieved limited commercial success, with only a handful of established companies and market-ready products. Advancing the field will require coordinated investment and collaboration among governments, entrepreneurs, investors, business leaders, academics, and engineers. At the same time, societal concerns – particularly around safety, ethics, and governance – continue to spark debate. To ensure responsible and sustainable growth, it will be essential to strengthen public awareness and education, and to develop comprehensive national and international regulations and standards (Fig. 1).

**Fig. 2:**
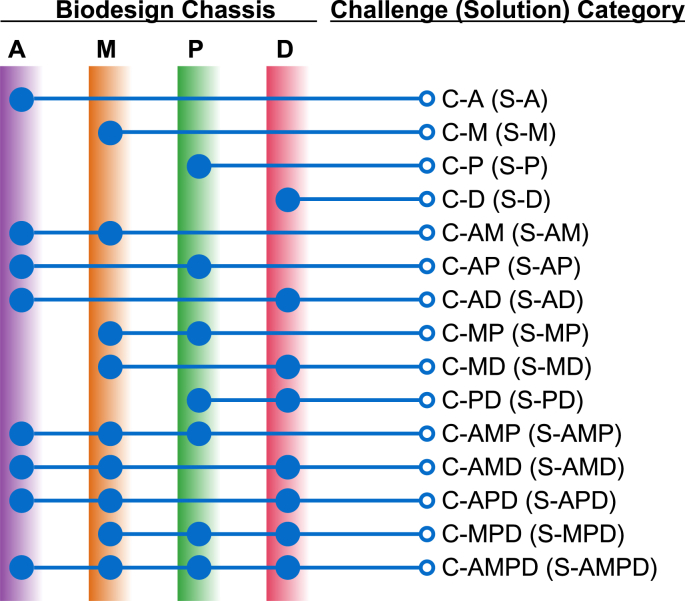
Classification of biodesign challenges and their associated solutions by chassis type. Challenges are labelled "C-chassis code(s)" and corresponding solutions (in parentheses) "S-chassis code(s)". The chassis codes are: A ​= ​Animal chassis, M ​= ​Microbial chassis, P=Plant chassis, and D ​= ​*de novo* chassis. Created with BioRender.com.

Biodesign challenges can be systematically categorized into 15 theoretical groups based on chassis types (Fig. 2). These challenges may be unique to specific chassis types (i.e., animal, microbial, plant, *de novo*) or shared across multiple types. This classification provides a structured framework for analyzing challenges and developing targeted solutions within biodesign research. Many challenges are specific to individual chassis types. For example, a major hurdle in animal biodesign is immune rejection in pig-to-human xenotransplantation, which significantly limits the viability of engineered animal organs as a solution to the shortage of human donor organs [3]. In the context of human biodesign, CRISPR-based gene therapy represents a transformative advancement, offering the potential to correct genetic disorders at their source. While promising, this innovation's adoption is limited primarily by its high implementation costs. CRISPR-based therapies, such as those targeting sickle-cell disease are projected to cost around $2 million per patient [4], highlighting the financial barriers to deploying cutting-edge technologies. Engineering microbes for applications ranging from therapeutics to biofuels is central to the bioeconomy. However, achieving stable and functional genetic circuits over relevant timescales remains a major challenge, largely because it is difficult to predict the required modifications a priori [5]. One challenge specific to plant biodesign is the genetic improvement of photosynthetic efficiency [6]. Among the four types of biodesign chassis, *de novo* chassis presents the most formidable obstacles. For example, assembling synthetic genomes and successfully initiating them within a cell or cell-like container to produce a viable organism remains a significant bottleneck [7].

In addition to chassis type-specific challenges, several significant hurdles are shared across multiple chassis types. One such challenge is the genotype dependency of genetic transformation, which affects plant, animal, and microbial chassis. While the underlying mechanisms differ – such as shoot regeneration from transformed cells in plants [6], innate restriction-modification systems in microbes that defend against foreign DNA [8], and DNA repair or integration mechanisms in animals [9] – the difficulty of achieving efficient and consistent transformation remains a consistent barrier. Another shared obstacle is the presence of biological trade-offs, where improving one trait often compromises another. For example, in plants, increasing yield may reduce stress tolerance [6,10]; in animals, boosting growth can weaken immunity [11]; and in microbes, maximizing product output may slow growth rates [12]. CRISPR-based genome editing is widely used across animal/human, microbial, and plant biodesign. However, off-target effects remain a significant challenge, limiting its broader applications in biodesign. Finally, all four biodesign chassis face the overarching challenge known as the "Valley of Death" - the crucial gap between promising, laboratory discoveries and commercially viable, real-world applications [13].

Realizing the full potential of biodesign to boost the bioeconomy, improve human health, enhance food security, and protect the environment depends on overcoming a range of inherent challenges. These challenges can be addressed through two broad categories of solutions: chassis type-specific and cross-chassis approaches, as shown in Fig. 2. To formalize the relationship between challenges and their corresponding solutions, we define a function f mapping each challenge to a set of weighted solutions:

(1) f:C⟶P(S×[0,1]),

where *C* ​= ​{*C*_1_, *C*_2_, …, *C*_m_} represents the set of all biodesign challenges, *S* ​= ​{*S*_1_, *S*_2_, …, *S*_n_} represents the set of all available solutions, and P(S×[0,1]) is the power set of solution-weight pairs, representing subsets of *S* with associated effectiveness weights ranging from 0 (no effectiveness) to 1 (fully effective).

**Fig. 3:**
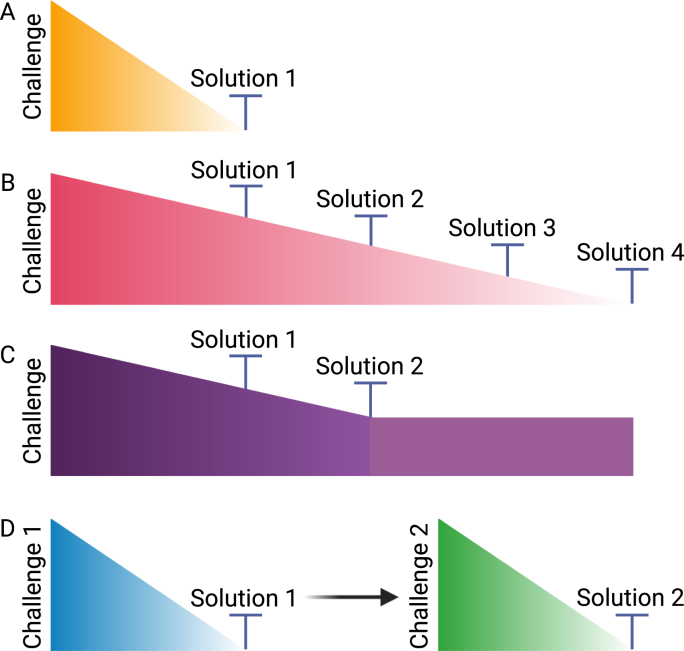
Dynamics of challenges and solutions in biodesign research. (A) A challenge is fully resolved by a single solution. (B) A challenge is fully addressed through multiple solutions. (C) A challenge is partially mitigated by multiple solutions. (D) Solving one challenge leads to the emergence of a new challenge, requiring additional solutions. Created with BioRender.com.

Specifically, the interactions between challenges and solutions can be classified into the following four types:

Type A: A challenge can be fully resolved by a single solution (Fig. 3A), defined as

(2) $$f\left(C_{i}\right)=\{(S_{j},1)\}$$

For example, lignin content plays a critical role in the utilization of poplar biomass. While genetically reducing lignin can enhance saccharification (the release of fermentable sugars), if often leads to undesirable growth defects. This challenge can be addressed through fiber-specific suppression of lignin production. By targeting lignin reduction specifically to fiber cells, researchers improved wood quality and sugar release efficiency without compromising overall plant growth [14].

Type B: A challenge can be fully resolved through the application of several coordinated solutions (Fig. 3B), defined as

(3) $$f\left(C_{i}\right)=\left\{\left(S_{j1},k_{i,j1}\right),\left(S_{j2},k_{i,j2}\right),...‚\;\left(S_{jn},k_{i,jn}\right)\right\},\text{with}\;\sum_{l=1}^{n}k_{i,jl}\geq 1$$

A key challenge in plant biodesign is the inherently low photosynthetic efficiency of most crops, which convert less than 1 ​% of available sunlight into stored chemical energy. Addressing this limitation requires a combination of strategies, including engineering ribulose-1,5-bisphosphate carboxylase/oxygenase (Rubisco), introducing carbon-concentrating mechanisms, and implementing synthetic photorespiratory bypasses [6].

Type C: A challenge can be partially mitigated by multiple solutions (Fig. 3C), defined as

(4) $$f\left(C_{i}\right)=\left\{\left(S_{j1},k_{i,j1}\right),\left(S_{j2},k_{i,j2}\right),...‚\;\left(S_{jn},k_{i,jn}\right)\right\},\text{with}\;0<\sum_{l=1}^{n}k_{i,jl}<1\text{.}$$

For example, despite decades of research and some notable progress, successfully engineering nitrogen fixation (N_2_ fixation) into non-leguminous crops like corn, wheat, and rice remains a major unresolved challenge [15].

Type D: Resolving one challenge leads to a new challenge (Fig. 3D), defined by an auxiliary function g that describes recursive interactions, mapping resolved challenges to newly emerging challenges:

(5) $$g:C\times S\;\to \;P\left(C\right),\text{such}\;\text{that}\;g\left(C_{i},S_{j}\right)=\{C_{new1},C_{new2},C_{new3},...\}$$

For example, While overexpressing the developmental regulators significantly improves transformation efficiency in various plant species, this approach can adversely affect the growth and development of the regenerated plants [6].

In conclusion, biodesign stands at the forefront of 21st-century science and technology, offering transformative or disruptive solutions across health, agriculture, industry, and environmental sustainability. However, realizing its full potential requires a deeper understanding of the complex challenges that exist both within individual chassis and across chassis boundaries. By systematically categorizing these challenges and illustrating their interdependence and solution pathways, this editorial presents a conceptual framework to guide future biodesign research and innovation. The proposed classification underscores the need for integrative, adaptive, and multidisciplinary strategies to address the diverse obstacles facing the field. A shared roadmap – one that incorporates both chassis-specific insights and cross-chassis synergies – is essential to accelerate progress. Such a roadmap will be key to unlocking biodesign's full potential to drive the bioeconomy, improve global health, and promote environmental sustainability. We call for papers that address chassis-specific or cross-chassis challenges in animal, microbial, plant, and *de novo* biodesign by proposing potential solutions or presenting transformative/disruptive research.

## Funding

The writing of this manuscript was supported by the U.S. Department of Energy, Office of Science, Biological and Environmental Research Program, Genomic Science Program, as part of the Center for Bioenergy Innovation (CBI) under FWP ERKP886 and the Secure Ecosystem Engineering and Design (SEED) Scientific Focus Area under FWP ERKPA17. Oak Ridge National Laboratory is managed by UT-Battelle, LLC for the U.S. Department of Energy under Contract Number DE-AC05-00OR22725. This work was also supported by USDA/NIFA grants – 2022-51300-38058, 2023-67015-39566 and 2023-67015-40080 to ZJ.

## Declaration of competing interest

The authors declare that they have no known competing financial interests or personal relationships that could have appeared to influence the work reported in this paper.
